# Examination of Sex‐dependent sleep disruptions and OSA severity on Limbic System Integrity in Community‐Dwelling Cognitively Unimpaired Older Adults

**DOI:** 10.1002/alz.092620

**Published:** 2025-01-09

**Authors:** Joshua L Gills, Oliver Cesar, Zanetta Kovbasyuk, Anhiti Dharmapuri, Julian McBride, Alfred Mbah, Elena Valkanova, Luisa F Figueredo, Ankit Parekh, Girardin A Jean‐Louis, Andrew Varga, Ricardo S. Osorio, Omonigho M Bubu

**Affiliations:** ^1^ NYU Grossman School of Medicine, New York, NY USA; ^2^ University of South Florida, Tampa, FL USA; ^3^ Mount Sinai School of Medicine, New York, NY USA; ^4^ University of Miami Miller School of Medicine, Miami, FL USA; ^5^ Icahn School of Medicine at Mount Sinai, New York, NY USA; ^6^ Center for Sleep and Brain Health, Department of Psychiatry, NYU Langone Health, New York, NY USA

## Abstract

**Background:**

Microstructure impairments of the limbic tracts are seen in early stages of the Alzheimer’s disease (AD) continuum. Sleep disruptions differ between sexes and have been linked with AD. We examined sex differences between measures of limbic white matter tracts and objective sleep parameters in cognitively unimpaired older adults.

**Method:**

This cross‐sectional study included 170 community‐dwelling cognitively unimpaired older adults (mean±SD: age=67.2±5.2y) participating in NYU studies of sleep, aging, and memory. Subjects completed polysomnography (NPSG) and brain MRI. Sleep measures of interest included total sleep time (TST), NREM stages 2, 3 and REM sleep duration, sleep efficiency and fragmentation, and AHI4% and REM AHI4%. Microstructural properties of the cingulum, uncinate fasciculus (UF), and fornix were estimated using diffusional tensor imaging (DTI) metrics including radial kurtosis (RK), radial diffusivity (RD), and fractional anisotropy (FA). Linear mixed‐effects regression models examined associations between sex*sleep variables and DTI metrics. Models were adjusted for age, race, education, BMI, time between NPSG and MRI.

**Result:**

Participants were 71.2% female, 44.1% Black/African‐American, and had 16.8±2.5y of education. In the cingulum, females showed increased fragmentation associated with decreased FA(β[IV]= β[SE}; β[fragmentation Left] =‐0.0023[0.00097], p=0.021), higher RD (β[fragmentation Right] =0.0021[0.00087], p=0.016; β [fragmentation Left] =0.0032[0.00096], p=0.001), and lower RK (β[fragmentation Right] =‐0.0055[0.00253], p=0.039). Additionally in the cingulum, females demonstrated higher AHI4% being associated with lower FA (β[AHI4% left]=‐0.0005[0.00026], p=0.054), higher RD (β [AHI4% left] =0.0006[0.00036], p=0.029), and lower RK (β [AHI4% Right] =‐0.0014[0.00069], p=0.052); and, less time spent in REM was associated with higher RD (β [REM left] =‐0.0016[0.00077], p=0.042). In the UF, females showed greater sleep fragmentation being related to higher RD (β[Fragmentation Left]=0.0045[0.00173], p=0.010) and lower RK (β[Fragmentation]=‐0.00077[0.00034], p=0.027), higher AHI4% was related to higher RD (β[AHI4% Right]=0.00072[0.00030], p=0.015). In the cingulum, men showed more NREM stage 3 sleep being associated with greater RK β[NREM 3 Left]=0.0065[0.00279], p=0.021). In the UF, men with higher AHI4% demonstrated associations with lower RK β[AHI4% Right]=‐0.0016[0.00080], p=0.046).

**Conclusion:**

In this sample of community‐dwelling cognitively unimpaired older adults. Sex‐specific sleep disruption associations were observed with limbic white matter tract alterations. Thus, highlighting possible sleep related underpinnings of sex differences in Alzheimer disease risk.

